# Wearable Assistive Robotics: A Perspective on Current Challenges and Future Trends

**DOI:** 10.3390/s21206751

**Published:** 2021-10-12

**Authors:** Uriel Martinez-Hernandez, Benjamin Metcalfe, Tareq Assaf, Leen Jabban, James Male, Dingguo Zhang

**Affiliations:** 1 Multimodal Inte-R-Action Lab, University of Bath, Bath BA2 7AY, UK; jjm53@bath.ac.uk; 2 Centre for Autonomous Robotics (CENTAUR), University of Bath, Bath BA2 7AY, UK; bwm23@bath.ac.uk (B.M.); ta608@bath.ac.uk (T.A.); dz492@bath.ac.uk (D.Z.); 3 Centre for Biosensors, Bioelectronics and Biodevices (C3Bio), University of Bath, Bath BA2 7AY, UK; lj386@bath.ac.uk; 4 Department of Electronics and Electrical Engineering, University of Bath, Bath BA2 7AY, UK

**Keywords:** wearable assistive robotics, wearable sensors, machine learning, human factors, standards

## Abstract

Wearable assistive robotics is an emerging technology with the potential to assist humans with sensorimotor impairments to perform daily activities. This assistance enables individuals to be physically and socially active, perform activities independently, and recover quality of life. These benefits to society have motivated the study of several robotic approaches, developing systems ranging from rigid to soft robots with single and multimodal sensing, heuristics and machine learning methods, and from manual to autonomous control for assistance of the upper and lower limbs. This type of wearable robotic technology, being in direct contact and interaction with the body, needs to comply with a variety of requirements to make the system and assistance efficient, safe and usable on a daily basis by the individual. This paper presents a brief review of the progress achieved in recent years, the current challenges and trends for the design and deployment of wearable assistive robotics including the clinical and user need, material and sensing technology, machine learning methods for perception and control, adaptability and acceptability, datasets and standards, and translation from lab to the real world.

## 1. Introduction

### 1.1. Importance of Wearable Assistive Robotics

**Figure 1:**
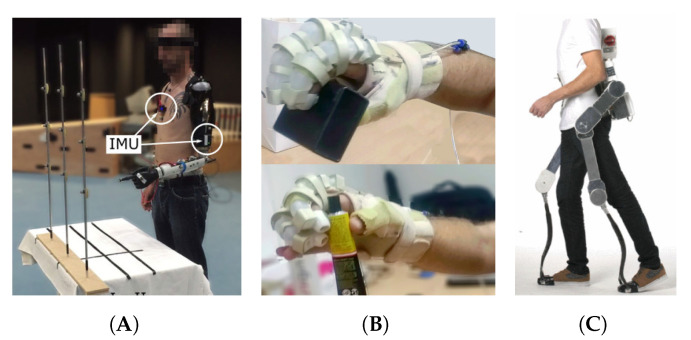
Wearable assistive robots such as (**A**) prosthetics [5] (Licensed under (CC BY 4.0)), (**B**) orthotics [6]) (Reproduced with permission from Elsevier) and (**C**) exoskeletons [8] (Licensed under (CC BY 4.0)), can help users to restore weak limb functions and substitute lost limbs.

Wearable assistive robotics has emerged as a promising technology to assist humans to enhance, supplement or replace limb motor functions, commonly affected after suffering an injury, a stroke or as a result of natural aging [1,2]. This robotic assistance is important to enable humans to perform physical and social activities of daily living (ADLs) independently, contributing to both dignity and improved quality of life [3,4]. Wearable robots can be found as exoskeletons, orthotics and prosthetics, capable of extending the strength of human limbs, restoring lost or weak limb functions and substituting lost limbs, respectively (Figure 1) [5,6,7,8,9,10,11,12]. These assistive devices are designed to be worn by humans and closely interact with the human body. Therefore, wearable robots need to be safe, reliable and intelligent, but also compliant, lightweight and comfortable to ensure the correct assistance, the safety of the user, and acceptability and usability of the device [13,14]. These requirements can be achieved by making use of technological advances such as multimodal wearable sensors, soft and hybrid materials, actuation systems, data fusion and machine learning and robotic architectures.

Section 2, Section 3 and Section 4 provide a description of the aspects involved in the design of wearable assistive robots including: the user perspective, methods for data processing and decision-making, actuators and materials for fabrication. Section 5 and Section 6 describe the challenges faced by current assistive technologies and forecast future trends in the field.

### 1.2. Clinical Need—Target User Groups

The world health organisation (WHO) estimates that 2 billion people will require assistive devices by 2050, doubling the current estimate [15]. This increase is driven by the growth of the aging population [16], people with upper and lower limb impairments, noncommunicable diseases and mental health conditions [17,18,19]. Wearable assistive robotic devices can enable their users to gradually recover the capability to perform ADLs independetly and naturally, leading a healthier life. Despite the importance of this technology, only 10% of those in the need of assistance have access to these robotic devices [15]. This limited access represents an issue for a sustainable future for all, which is one of the Sustainable Development Goals identified by the United Nations (UN) that strive to “leave no-one behind” [20,21]. The Global Cooperation on Assistive Technology (GATE) is another initiative created to improve global access to assistive devices [22], including wearables such as fall detectors, hearing aids, lower-limb prostheses and talking and touching watches [23].

The spread of smart and wearable technologies offers a strong set of tools to develop wearable assistive robots [24] that can impact positively on physical and social aspects of users [25]. Wearables also have an advantage over other forms of assistive technologies (e.g., handheld devices, mobility aids and distributed systems [26]) in their continuous close proximity to the user and compliance to the human body. These aspects enable the systems to collect vital data to provide customised assistance highly valued by people with different levels of physical, sensory and cognitive impairments [25,27].

## 2. Wearable Assistive Robots Technologies

Wearable assistive robots are designed with the goal to assist humans with physical impairments, particularly, assisting lower and upper limbs, and joints on the human body. These robots, which work in proximity with the human body, can be built using different material technology usually composed of rigid, soft or hybrid materials.

### 2.1. Rigid Materials in Wearable Robotics

**Figure 2:**
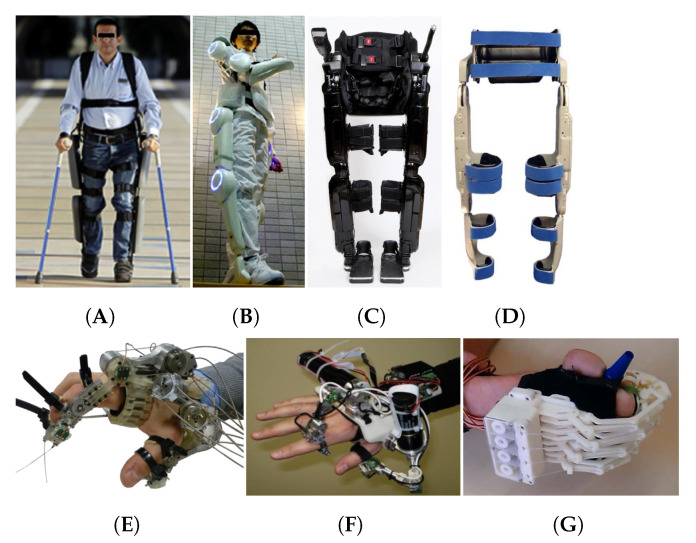
Wearable assistive robots with rigid and semi-rigid structure. Lower limb assistive robots: (**A**) ReWalk [12] (Reproduced with permission from Elsevier), (**B**) HAL [28] (Licensed under (CC BY 2.0)), (**C**) REX [32] (Licensed under (CC BY 4.0)), (**D**) Vanderbilt exoskeleton [33] (Reproduced with permission from Elsevier). Wearable assistive hands: (**E**) HandeXos-Beta [34] (Reproduced with permission from Elsevier), (**F**) HexoSYS [35] (Reproduced with permission from Elsevier), (**G**) HES Hand [36] (Reproduced with permission from Elsevier).

Rigid and semi-rigid materials have been used traditionally for the development of exoskeletons, widely employed for assistance in locomotion activities. The ReWalk robotic system with a semi-rigid structure can assist the knee and hip of adults with partial and complete mobility impairments [12], detecting and enhancing the user’s walking action. Vanderbilt, REX and HAL are other examples of these type of assistive robots built with rigid structures (Figure 2). They can assist humans to keep balance while walking but also to accomplish daily activities including sitting, climbing stairs and kneeling [28,29,30,31,32,33]. Wearable robot hands can assist with daily activities such as buttoning, grasping, pouring liquids, closing and opening zips and jars. HandSOME, HandeXos-Beta, HexoSYS, HES Hand and Vanderbilt Hand are wearable hands with rigid structures that use, arrangements of motors and tendons to provide the required assistance (Figure 2) [34,35,36,37,38,39]. These lower and upper limb devices can be configured to respond quickly to the user movement intention using data from electromyographic (EMG), inertial measurement unit (IMU) and torque sensors. Unfortunately, these devices tend to be bulky, heavy (lower limb robots weighing between 15 kg to 25 kg) and expensive. The rigid structure of these robots can also make them uncomfortable and constraining the natural movement of human limbs in certain orientations.

### 2.2. Soft Materials in Wearable Robotics

**Figure 3:**
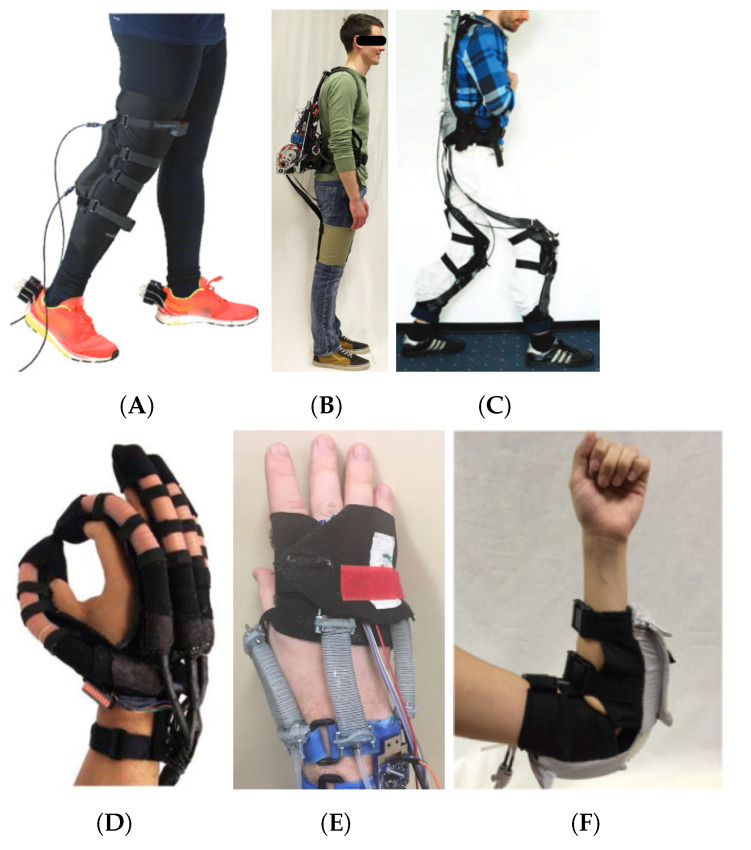
Wearable assistive robots with soft structure. Lower limb assistive robots: (**A**) soft-inflatable knee exosuit for rehabilitation [42] (Licensed under (CC BY 4.0)), (**B**) soft exosuit for hip assistance [44] (Reproduced with permission from Elsevier), (**C**) multi-articular hip and knee exosuit [46] (Reproduced with permission from Elsevier). Upper limb assistive robots: (**D**) soft robotic glove for assistance at home [47] (Reproduced with permission from Elsevier), (**E**) soft wearable wrist for rehabilitation [48] (Licensed under (CC BY 4.0)), (**F**) soft robotic elbow sleeve for assistance [49] (Licensed under (CC BY 4.0)).

Soft materials are becoming popular in the area of assistive robotics with different system developed in recent years to assist upper and lower limbs (Figure 3) [40,41]. Assistive robots with soft materials tend to be lighter and more comfortable compared to robots built with rigid structures. Pneumatic artificial muscles, Boden cables, textiles and shape memory alloys are the main material technologies that have been employed in a variety of wearable soft robots. Soft wearable knee and ankle robots have been used to assist contraction/extension leg movements and also with foot movements in dorsiflexion, plantarflexion, inversion and eversion orientations [42,43]. Exosuits using textiles and Boden cables are some of the most advanced and lightweight devices for assistance to the hip, leg and ankle-foot while walking on flat surfaces [44,45,46]. This technology has been explored with soft gloves, wrist and elbows for reaching, grasping, holding and manipulating objects, but also to potentially assist with buttoning and feeding (holding cutlery) [47,48,49,50]. Modular and customisable systems capable of adapting to the users body and required assistance have been investigated with designs based on tendons, shape memory alloys and pneumatic technology [51,52,53]. Soft materials offers a promising approach for wearable assistive robots that are comfortable, lightweight and do not constrain the movement of upper and lower limbs. However, soft assistive robots still require external large gearboxes that affect the portability of the systems, and pumps that can slow down the system response. Table 1 shows the comparison of the wearable assistive robot technologies presented in this section based on their applications, fabrication materials, degrees of freedom (DoF), body segment assisted, actuation type and weight.

## 3. Wearable Sensing Technologies

**Figure 4:**
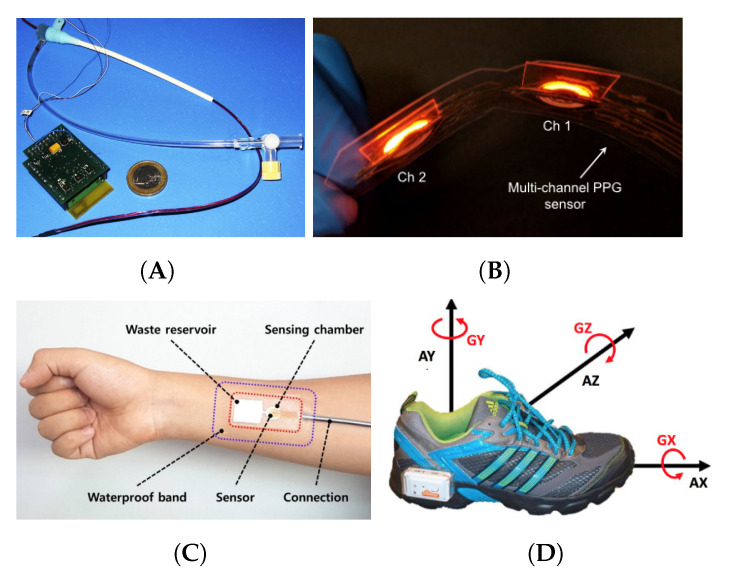
Wearable sensors. (**A**) Zigbee to monitor physiological parameters [54] (Reproduced with permission from Elsevier). (**B**) Optoelectronic wrist sensor for health monitoring [58] (Licensed under (CC BY 4.0)). (**C**) Stretchable electrochemical sensor for glucose monitoring [69] (Reproduced with permission from ACS). (**D**) On-shoe wearable sensor for gait analysis in Parkinson’s Disease [64] (Licensed under (CC BY 4.0)).

Rapid progress in the development of flexible electronics and materials has enabled the development of advanced wearable sensors for monitoring the state of the human body and the assistive robot. Some of the state-of-the-art wearable sensors can be found as e-textiles (e.g., smart garments) and e-patches (e.g., sensor patches) to monitor aspects such position and orientation of human limbs, human motion and muscle activation [54,55,56,57], heart function [58,59,60,61], brain activity, sleep apnea [62,63], Parkinson disease [64,65,66,67,68]; chemical and electrochemical sensors [69,70,71]. Examples of these sensors are shown in Figure 4. Many sensors have been used in healthy people to monitor, for example, sport activities as well as people affected by health conditions or for clinical tests to detect underlining conditions that could become more serious providing powerful diagnosis tools. These wearable sensors have also been used in devices such as ankle-foot robot orthotics, exosuits, soft wearable gloves and hand exoskeletons to assist humans to rehabilitate and perform ADLs. Another interesting aspects of wearables is that many of these devices can be used by care givers to monitor patients [72,73] to detect and manage treatments or conditions at home [74,75], which have had a direct impact on quality of life and influence on the care sector. Wireless communication technologies, lower power electronics, soft materials and the internet of things (IoT) [76] have largely helped wearable sensors to become smaller, lighter and less cumbersome (e.g., no/less wires), but also to make the sensor data accessible remotely for online monitoring, processing and control.

## 4. Machine Learning in Wearable Assistive Robots

Machine Learning (ML) offers a promising approach, capable of perceiving and making decisions, to develop intelligent wearable robots that can safely and accurately assist humans. This section provides a brief description of ML methods and Deep Learning (DL) based approaches for activity recognition with different wearable sensor platforms.

### 4.1. Traditional Machine Learning Methods

Traditional ML methods are generally well understood and can provide state-of-the-art results primarily during training. This approach includes methods such as Support Vector Machines (SVM), Random Forests (RF), Bayesian Inference, Decision Trees (DT), k-Nearest Neighbour (kNN) and logistic regression. Recognition of hand gestures has been studied using wearable sensors (bending and force sensors) and SVM, kNN and DT methods. SVM achieved the highest recognition accuracy in real-time mode, although it required the longest training and prediction time compared to kNN and DT methods [77]. SVM has also outperformed kNN, logistic regression and RF methods for recognition of grip action from an assistive tactile arm brace (TAB) worn on the forearm of participants [78]. However, in human activity recognition (HAR) with IMU data, RF methods showed to perform better compared to SVM, DT, NN, kNN and Naive Bayes methods [79]. RF methods have also shown accurate results for HAR with datasets containing IMU, audio and skeletal data [80]. Bayesian methods and sequential analysis have achieved accurate recognition of sit-to-stand activities using a single wearable accelerometer [81]. The combination of traditional machine learning methods and DL, such as Hidden Markov Models (HMM) and NN, has ensured reliable prediction of sequential gait stages [82]. Data-driven and Fuzzy Logic approaches are model-free and make use of knowledge from experts, they have been used for classification and system control [83,84], and recently in the recognition of ADLs and control of wearable assistive and rehabilitation robots [85,86,87].

### 4.2. Deep Learning

Deep Learning methods are primarily focused on Neural Network (NN) based architectures, and have demonstrated their ability for accurate activity recognition with complex datasets where other machine learning methods fail [88]. This section briefly presents three DL architectures widely used: the Multilayer Perceptron (MLP), Convolutional Neural Network (CNN) and Recurrent Neural Network (RNN).

**Figure 5:**
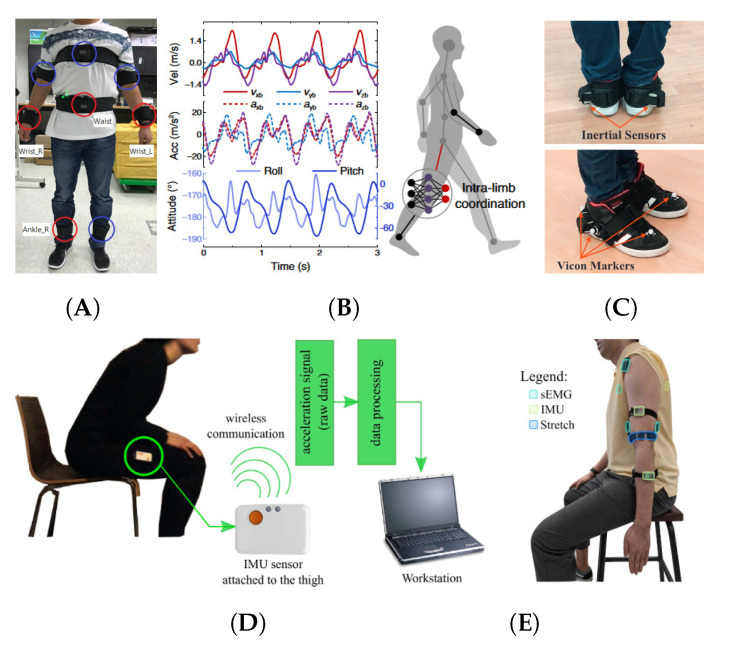
Examples of wearable systems that exploit the use of machine learning methods for activity recognition needed for assistive robots. (**A**) Multimodal sensor fusion with Long Short Term Memory networks for recognition of walking, sitting, lying, standing, driving and eating [103] (Licensed under (CC BY 4.0)). (**B**) Artificial Neural Networks for recognition of dynamic motion of human limbs [89] (Licensed under (CC BY 4.0)). (**C**) Gait detection using multimodal sensors with Hidden Markov Models and Artificial Neural Networks [82] (Reproduced with permission from Elsevier). (**D**) Detection of sit-to-stand and stand-to-sit using IMU sensors and Dynamic Bayesian Networks [81] (Reproduced with permission from Elsevier). (**E**) Prediction of elbow motion using multimodal sensors with Random Forest and Decision Trees [104] (Licensed under (CC BY 4.0)).

Multilayer Perceptrons were applied to data from accelerometers, gyroscopes and velocity sensors on the shank of users to predict the attitude angles of the thigh for walking assistance [89]. MLPs can recognise activities with IMU data [90], however, CNN and RNN methods have achieved higher accuracy for the recognition process. Surface electromyography (sEMG) sensors alongside MLPs and Linear Discriminant Analysis (LDA) have been used to recognise hand gestures and perform better than SVM [91]. CNNs have become popular for feature extraction from complex datasets for classification and recognition tasks. Image recognition and segmentation are key areas of CNNs applied to wearable devices for assistance to people suffering from visual impairment. FuseNet and GoogLeNet architectures are other examples of DL methods for semantic scene segmentation in human auditory assistance to avoid obstacles [92]. CNN architectures and IMU data have shown extensive use in HAR tasks and have achieved improved results when compared to other traditional methods [93,94,95,96,97]. RNN methods are becoming widely used for tasks that require the analysis of sequential data or events. Examples being the use of a Long Short Term Memory (LSTM) backend on the DeepConvLSTM architecture [98,99] or InnoHAR, an inception based CNN architecture followed by two Gated Recurrent Unit (GRU) layers [100]. Recent developments in the use of attention based models have shown their potential for accurate labelling of multimodal wearable sensors data, such as data from HAR tasks performed with upper and lower limbs [101,102]. Figure 5 shows examples of the use of LSTM, ANN, HMM, DBN, RF and DT methods for activity recognition with different wearable sensor platforms [81,82,89,103,104].

### 4.3. Sensor Fusion

Although sensor fusion is not an ML method, it is a process that can provide a better representation of input data for the subsequent ML processes, and improve the certainty, accuracy and completeness over the results when using a single sensor [105]. The fusion process can be classified as competitive, cooperative or complementary. The competitive fusion approach refers to multiple sensors to measure the same feature, while complementary fusion refers to measuring different aspects of the same phenomenon. Cooperative fusion refers to sensors measuring different attributes, where all are required to form a complete understanding of what is measured [106,107].

Various fusion methods have been studied with wearable sensor data and ML methods. A stacked generalisation architecture using RF first learner and meta learner classifiers was used for the fusion of audio and accelerometer data while performing ADLs [80]. A similar architecture using LSTM first learners on IMU data showed an RF meta learner to outperform SVM and kNN methods, however, a soft voting approach provided higher accuracy yet. The fusion of RF methods outperformed the use of a single RF method when recognising heart disease with a variety of body worn sensors [108]. Fusing IMU and vision as input data to a CNN first learners, and an LSTM fusion method, showed to outperform a variety of methods for the recognition of upper limb actions for assembly tasks in an industrial setting [109]. Fusion of acceleration and angular velocity data images with a CNN ResNet fusion classifier achieved higher recognition accuracy over other methods such as sum, average and maximum [110]. Gesture recognition was improved by the fusion of wearable pressure sensors and radar data from the finger movements with an SVM method [111].

## 5. Current Challenges in Wearable Assistive Robots

Wearable assistive robotics still faces various challenges to ensure that these systems are reliable, functional in real environments and comply with the user needs. These challenges, which include device adaptability, translation from the lab to the real world, sensing technology and user acceptability, are discussed in this section.

### 5.1. Device Adaptability to the User (Personalised Robotics)

The need for personalised and adaptive assistive devices is widely recognised. Adaptivity refers to how devices adapt to changes within their operating environment, such changes might include: user habits, situations, individual preferences and exogenous changes. In practice adaptivity means changing the way of the assistive robot to respond to specific user commands, detection of user intent, reacting to exogenous changes such as sensor instability, or modification of the intervention modality [112]. These changes can be split into two broad categories: user driven and exogenous, which require the use of contextual information together with a model of the user or system to ensure the correct adaption process. Adaptivity is often viewed as a machine learning problem, with much work on Human Activity Recognition (HAR) making use of supervised learning techniques to provide the vital contextual awareness [113]. Activities are often identified by comparing sensor events over sliding time windows with templates that are fit to specific ADLs, these templates can be iteratively developed but are generally fixed and are used to drive an ontological model [114]. These model-based approaches lack the flexibility required to allow wearable assistive robots to deal with the level of changes that might be expected in outdoor environments and may not accurately capture the complexities or diversity of many use cases. Hybrid solutions exist, where data and knowledge (activity recognition and model based) approaches are combined to form hybrid solutions [115]. Initial knowledge can be used to form templates that enable initial activity detection, while newly discovered activities are recorded for later labelling, often with active participation of the user. More recently, Smith et al. have proposed the Adaptivity as a Service (AaaS) approach that encompasses all the dimensions of adaptation. AaaS addresses the three types of long-term adaptation: contextual awareness, adaptation in intervention modality, and rapid adaptation to new users [112]. Various works have proposed methods to address the adaptivity of assistive systems, however, it remains a current challenge to ensure safe, reliable and efficient robot assistance and device acceptability.

### 5.2. Translational Issues from the Lab to the Real World

Wearable assistive robots are usually designed and tested in labs under well-controlled conditions and structured environment. The testing processes tend to follow protocols designed for the lab only, where the user performs actions in a predefined order, controlled setting and as indicated by the researcher. For instance, walking along a certain circuit composed of flat surfaces, stairs and ramps, grasping objects and moving them from one point to another [116,117]. In lab settings, these experiments involve the use of treadmills with controlled speeds and inclinations, and objects specifically designed for manipulation. This approach allows the systematic data analysis, monitoring and control of the assistive device while responding to different user movements. However, this process still does not reflect the situations than a person can experience in outdoor environments, such as walking on different terrains and condition environment, changing the walking speed, grasping and handling real objects for daily use [118]. This difference in testing settings affects the robot performance for sensing, making decisions and controlling the assistance required by the user. This translation of assistive robots from the lab to real environments represents an important issue that needs to be addressed to have robots that can be accepted and daily used by individuals. Computational methods are also generally designed and trained assuming the availability of clean and accurate data from wearable sensors and the robotic system [86]. This assumption works very well in simulation and well-controlled laboratory environments, however, their performance is drastically affected when tested in real environments. Furthermore, unexpected, different and continuous changing of daily situations experienced by the person undoubtedly generate unseen complex and unlabelled data decreasing the performance of the assistive robots and putting in risk the safety of the user [119,120]. Therefore, it is important to have systems with methods capable of learning continuously from the state of the human body, robot and environment to adapt to daily and unexpected situations safely and accurately.

### 5.3. Wearable Sensing Technology

Even though sensor technology has advanced rapidly allowing multimodal monitoring capabilities in wearable assistive devices, there are elements such as low-weight, low-power consumption, battery lifespan and calibration that remain a challenge. Having sensing devices with low-power consumption can extend the monitoring time between charges, help to reduce the weight needing smaller energy storage, but also it can open opportunities to exploit emerging technology such as energy harvesting [121] directly from the user and wearable robot e.g., while performing locomotion activities. Battery lifespan, weight, and maintenance are major elements to be factored in the process of sensor design. These aspects enable assistive robots to be used for longer periods without recharging the battery but also without the need to calibrate the sensors or replace them. Currently, there are also many elements not directly related to materials and new technologies that play a major critical role in wearable technologies with major challenges. Some of these are communication modules (e.g., WiFi, Bluetooth), data integrity and protection, difficulties in precise localization using for example GPS and mobile-phones [122] and design of suitable interfaces to allow information extraction from users that could have disabilities or difficulties to interact with digital systems.

These technical challenges need to be addressed for the design of wearable sensing technology. However, there is a lack of robust, systematic and testing on large number of patients with different requirements for monitoring and assistance that represent a bottleneck for the use of wearable sensors in a wide range of monitoring and assistive systems [123,124]. This process is usually limited or even ignored due to the time and cost of recruiting patients, preparing and running the tests, and thus, limiting the design of wearable sensors to academic and well-controlled environments only. The understanding of this sensor-patient interface, by keeping patients in the loop, is essential to develop wearable sensors that are clinically viable and approved to run under real conditions and patients but also robust, reliable and comfortable for the user [125].

### 5.4. User Acceptability

Even with the technological advances in assistive technology, device abandonment remains one of the main challenges. Upper-limb prostheses can be taken as an example where up to 75% of the users reject their prosthesis [126], given that they find the device uncomfortable and insufficiently functional [127]. Acceptance studies show similar trends across different assistive technologies [128], where the reasons behind device abandonment vary between users but tend to emphasise the mismatch between expectations and reality [129,130]. These issues have motivated researchers to move towards a more user-cantered design process. Co-creation is a design framework that has gained interest recently [131,132,133,134]. It ensures the participation of different stakeholders to minimise the mismatch between user needs and the developed product features and maximise impact. Stakeholders included are the users, healthcare professionals as well as industrial representatives and policy makers. Each of the stakeholders provide a special perspective. The users are at the heart of the process enabling the designers to gain empathy and learn from the users’ lived experience [135]. Healthcare professionals provide a more holistic perspective of common concerns and issues that arise. Their involvement is particularly important as they guide and support users during the decision making process of assistive technology [136]. Investors and policy makers are key to ensure that the translation of research does not end in what has been described as the “valley of death” that creates a gap between the available technologies and commercial products [137]. Weight reduction of the wearable assistive robot is another challenging aspect to make the system comfortable and acceptable for the user. Weight and portability are affected by heavier power supply systems, which are needed to provide longer autonomy as well as comply with activity-dependant assistive forces requirements, e.g., walking, running, sit-to-stand [138,139]. Some works have proposed the use of a compact variable gravity compensation approach to generate torque employing a cam and lever mechanism to improve the energy efficiency of assistive systems, surgical and mobile robots [140]. Spring-based gravity compensation mechanism and arrangement of springs have been used to improve energy efficiency for the delivery of fixed torques [141,142]. The Mechanically Adjustable Compliance and Controllable Equilibrium Position Actuation (MACCEPA) mechanism has shown to be an energy efficient actuation system for ankle-foot assistive robots and gait rehabilitation [143,144]. Recently, a design optimisation method, that uses mechanism parameters and mechanism architecture, has been employed to obtain the optimal arrangement of actuators to improve the energy efficiency of soft lower limb exoskeletons [138]. These works have shown impressive progress for actuation systems to assist with specific activities. However, the development of simple and energy efficient actuation systems, which would enable the use of small power supplies, remains a challenge in wearable robotics for assistance to ADLs.

Other important aspects to consider for the acceptability of the device is the user’s perception about the risk of using AI and IoT for data collection and sharing, and control of the system. These aspects make the user aware of ethical, privacy and safety concerns, which are more common amongst younger people- the future users of assistive devices currently being developed [145]. These concerns should be considered during the design process of assistive robots to ensure that individuals are able to trust and use the devices. Also, researchers need to be familiar with frameworks such as the Technology Acceptance Model (TAM) and Transparency paradigm to ensure that the relevant acceptance factors are included in the robot and to minimise the perceived risks of safety and privacy from AI and IoT [146,147,148,149].

## 6. Forecasting Future Trends on Wearable Assistive Robots

Undoubtedly, the field of wearable assistive robotics has seen impressive achievements given the advances in elements such as sensor technology, fabrication materials, machine learning and control methods, and computational power. However, to reach the aim of having smart and comfortable wearable systems that can assist humans to perform daily activities independently in a natural way, safely and efficiently, there are still aspects such as materials and sensing, learning and adaptability, datasets and standards that need to be considered and improved for the future development of wearable assistive robotics.

### 6.1. Hybrid Wearable Assistive Systems

Currently, most of the assistive robots are built using rigid or soft materials. Rigid materials have been widely used for the development of systems that can help humans to perform daily activities, but also to enhance the human strength to carried out tasks in industry. Soft materials has been gaining more attention in recent years for the development of wearable robots given that these materials are lightweight, compliant and do not constrain the natural movement of the human body, which usually occurs with rigid materials. Despite these characteristic they cannot provide the required torque to assist the human, for example lifting the legs for locomotion activities. Hybrid approaches, composed of rigid and soft elements, can offer a better trade-off for the design of assistive robots [150,151]. This approach can make the robot structure lightweight and capable of adapting to the human body, while still delivering the required torque safely [152]. This type of robot design enhances the sensing modalities and technology that can be integrated within the hybrid structure [153,154]. This can provide richer and larger datasets that have the potential to increase the repertoire of opportunities for research on novel machine learning and control methods for learning and adapting the level and type of required assistance [155,156]. Hybrid approaches, embedded with a large variety of sensing technologies, is a key aspect that we expect to see in the development of wearable assistive robots.

### 6.2. Learning and Adaptability to the User

Two key components required for the development of robots that can safely and efficiently assist humans are the capability to learn and adapt to the user over time [157,158]. Current wearable assistive robots are designed to support users under specific and controlled conditions, for example, assisting to walk and sit-to-stand or reach and grasp an object in well-controlled laboratory settings. These systems tend to fail when they are tested in outdoor environments or even when there are slightly changes in the test settings. For this reason, it is important to research and develop methods that allow the design of robots capable of learning and adapting to the user and changes in the surrounding environment. Advanced intelligent robot architectures, for data processing at different levels of abstraction, offer an approach to develop safe and reliable assistive systems, as has been seen in other robotic applications [159,160,161]. Usually, these architectures include modules such as sensing, data fusion, perception, decision-making and control, but also memory modules and reactive and adaptive layers for continuous learning and adaptive processes [162,163]. This approach can be implemented in wearable assistive robot architectures, together with novel machine learning and control methods, for example to allow the robot to identify the activity performed by the human, and thus deliver the required assistance. Another key aspect to achieve adaptability in the design of future assistive systems is the context of the task or activity [164,165]. Having wearable robots that for example know whether the person is at home or at work allow the system to identify the most likely actions that the person would perform, and thus, increase the reliability of decisions and assistive actions made the by robot [166]. This approach offers the potential to have smart wearable assistive robots that can safely react to unseen or unexpected events or data, but also to adapt to different and changing surrounding environments.

### 6.3. Datasets and Standards

Significant progress achieved in recent decades in robotics can offer huge societal benefits, however, they must be appropriately regulated and ethically developed. Datasets and standards are two key pillars in the ethical development of new robots. At present, significant research is undertaken purely because of dataset availability, rather than on a strong unmet clinical need (there is often poor correlation between clinical need and dataset availability). There are several publicly available datasets for ADL [167,168], however, they are usually collected and prepared using different protocols complicating analysis and replication. It is critical to have standards for robust and reliable collection and preparation of datasets correlating to the clinical needs, but also enabling researchers to replicate the data collection and analysis. Likewise, it is important that the emerging ethics are strongly linked to the development of standards and the implementation of regulations [169]. Current ethical frameworks include the EPSRC Principles of Robotics [170] and the 2006 EURON Roboethics Roadmap [171], and recent standards include ISO 13482 (Safety requirements for personal care robots) [172] and BS 8611:2016 (Robots and robotic devices in general) [173]. The IEEE has set forth on an ambitious programme of standards under the banner of the IEEE P7000 series, including standards on Data Privacy Processes (P7002), Ontological Standard for Ethically driven Robotics and Automation Systems (P7007) and IEEE Recommended Practice for Assessing the Impact of Autonomous and Intelligent Systems on Human Well-Being (Std 7010-2020) [174].

The combination of human factors with machine learning, sensors, materials and clinical feedback is crucial for the development of smart, efficient and comfortable wearable assistive robotics for daily usage by patients. The future development of this type of robot looks promising with great achievements and benefits for society in the coming years.

## 7. Conclusions

Wearable assistive robots have the potential to offer new alternative platforms and ways to deliver assistance, rehabilitation and care to users. This perspective paper has presented a list of design requirements to develop reliable, efficient, safe and comfortable assistive devices that can be used on a daily basis by patients. It has been shown that low-weight, portable and easy to put on and take off robots are key aspects to make robots comfortable for users. Soft robots tend to be lighter and more comfortable than rigid structures, however they still cannot deliver the required assistive forces, which suggests that the optimal approach is a hybrid approach with energy efficient actuation and control systems. Sensing technologies and computational methods have shown impressive progress for multimodal data collection and recognition of ADLs under well-controlled environments and for specific group of activities. Computational methods still have the challenge to perform reliably in daily life environments and respond safely to unseen data and unexpected body motions. This suggests that assistive systems need to be designed with the capability to learn continuously and adapt to the user and terrains autonomously. These aspects of autonomy and learning will require appropriate regulations for robust collection of datasets, privacy in data sharing and ethical design of assistive robots, but this will also require the direct involvement of clinicians and patients in the design process. All these aspects will ensure the development of reliable, safe and comfortable assistive robots, that are transparent in the decisions made and assistive actions performed, and thus, make the user trust the robot and accept it for daily usage.

## Figures and Tables

**Table 1:** Wearable robot technology for assistance to lower and upper limbs.

Assistive System	Application	Material Structure	Degrees of Freedom (DoF)	Assisted Body Segments	Actuation Type	Weight
ReWalk [12]	Assistance to stand upright and walk	Rigid materials	6 DoF	Hip flexion/extension Knee flextion/extension	Electric motors	23 kg
HAL [28]	Human gait rehabilitation, strength, augmentation	Rigid materials	4 DoF	Hip flexion/extension Knee flextion/extension	Electric motors	21 kg
REX [32]	Human locomotion in forward and backward directions, turn and climb stairs.	Rigid materials	10 DoF	Hip flexion/extension Knee flexion/extension Posture support	Electric motors	38 kg
Vanderbuilt exoskeleton [33]	Assistance for walking, sitting, standing, walking up and down stairs	Rigid materials	4 DoF	Hip flexion/extension Knee flexion/extension	Electric motors	12 kg
HandeXos- Beta [34]	Hand motion rehabilitation for multiple grip configurations	Rigid materials	5 DoF	Index finger flexion/extension Thumb finger flexion/extension and circumduction	Electric motors	0.42 g
HexoSYS [35]	Hand motion rehabilitation	Rigid materials	4 DoF	All fingers flexion/extension and abduction/adduction	Electric motors	1 kg
HES Hand [36]	Hand motion rehabilitation to recover hand motor skills	Rigid materials	5 DoF	All fingers flexion/extension	Electric motors	1.5 kg
Soft-inflatable knee exosuit [42]	Gait training for stroke rehabilitation	Soft pneumatic materials	1 DoF	Knee flexion	Pneumatic system, inflatable actuators	0.16 kg
Soft hip exosuit [44]	Assistance for level-ground walking	Soft textile materials	1 DoF	Hip extension	Electric motors, fabric bands	0.17 kg
Multi-articular hip and knee exosuit [46]	Assistance to gait impairments in sit-to-stand and stair ascent	Soft materials and Bowen cables	1 DoF	Hip and knee extension	Electrical motors, Bowden cables	-
Soft robotic glove for assistance at home [47]	Assistance to hand rehabilitation for grasping movements	Soft elastomeric chambers	3 DoF	All fingers flexion/extension	Pneumatic system	0.5 kg
Soft wearable wrist [48]	Assistance for rehabilitation of writs movement	Soft reverse pneumatic artificial muscles	2 DoF	Wrist flexion/extension and abduction/adduction	Pneumatic system	-
Soft robotic elbow sleeve [49]	Assistance for rehabilitation of elbow movements	Elastomeric and fabric-based pneumatic actuators	2 DoF	Elbow flexion and extension	Pneumatic system	-

## Data Availability

Not applicable.
